# Phylogenetic Analysis and Pathogenicity Assessment of Two Strains of Avian Influenza Virus Subtype H9N2 Isolated from Migratory Birds: High Homology of Internal Genes with Human H10N8 Virus

**DOI:** 10.3389/fmicb.2016.00057

**Published:** 2016-02-29

**Authors:** Ge Ye, Chai Hong Liang, Deng Guo Hua, Lei Yong Song, Yang Guo Xiang, Chen Guang, Chen Hua Lan, Hua Yu Ping

**Affiliations:** ^1^College of Wildlife Resources, Northeast Forestry UniversityHarbin, China; ^2^Harbin Veterinary Research Institute, Chinese Academy of Agriculture SciencesHarbin, China; ^3^Hubei Province Wildlife Epidemic Disease CenterWuhan, China

**Keywords:** influenza virus, H9N2, H10N8, H7N9, migratory birds, phylogenetic analysis, pathogenicity

## Abstract

Two human-infecting avian influenza viruses (AIVs), H7N9 and H10N8, have emerged in China, which further indicate that the H9N2 subtype of AIVs, as an internal gene donor, may have an important role in the generation of new viruses with cross-species transmissibility and pathogenicity. H9N2 viruses that contain such internal genes widely exist in poultry but are rarely reported in migratory birds. In this study, two strains of the H9N2 virus were isolated from fecal samples of migratory birds in 2014: one strain from Caizi Lake in Anhui Province and one from Chen Lake in Hubei Province of China. Nucleotide sequence analysis revealed high homology of all six internal genes of these two strains with the internal genes of the human H10N8 virus in Jiangxi Province, as well as with the human H7N9 virus. Phylogenetic analysis indicated a possible origin of these two strains from poultry in South China. Both of the two viruses tested could replicated in respiratory organs of infective mice without adaption, by both strains of the H9N2 AIVs from wild birds, suggesting their potential capacity for directly infecting mammals. Our findings indicate the existence of H9N2 viruses that contain internal genes highly homologous with human H10N8 or H7N9 viruses. Wild birds can contribute to the spread of the H9N2 virus that contains the “harmful” internal gene complex, leading to gene rearrangement with other influenza viruses and to the generation of new pathogenic viruses. Therefore, strengthening AIV surveillance in wild birds can promote an understanding of the presence and prevalence of viruses and provide scientific evidence for the prevention and control of AIVs and human-infecting AIVs.

## Introduction

The influenza virus poses a great threat to the animal breeding industry, wildlife protection, and human health on the global scale. Due to the continuous and unpredictable emergence of novel pathogenic subtypes or genotypes, this threat is becoming more serious and long lasting. Since 1996, highly pathogenic (HP) avian influenza virus (AIV) subtype H5N1 has prevailed widely and persistently in the world, becoming one of most harmful public health problems (Xu et al., 1999). The nonlethal influenza viruses also should not be negligible, they circulated in avian species can cause disease and even death in humans. In March and December 2013, China reported human deaths due to the novel AIVs H7N9 and H10N8 (Kageyama et al., 2013; Chen et al., 2014; Dai et al., 2014). H6N1 subtype AIVs was isolated from human with influenza-like clinical symptoms in Taiwan, 2013 (Yuan et al., 2013).

The H9N2 AIVs widely exist in wild birds and poultry in all over the world (Li et al., 2014) and some cases of human' infection (Peiris et al., 1999; Butt et al., 2005; Guo et al., 2005; Zhang et al., 2013a; Huang et al., 2015). Numerous studies have highlighted the importance of H9N2 AIVs in a potential influenza pandemic (Li et al., 2014; Yu et al., 2014; Sun and Liu, 2015), especially their role as the donor of an internal gene complex is of particular concern. H9N2 AIVs not only are the donors of internal genes for the HP H5N1 AIV found in Hong Kong in 1997 but also provide internal genes for the novel human H7N9 and H10N8 AIVs (Cui et al., 2014).

H9N2 viruses carrying such an internal gene complex are widely present in poultry, but they are rarely reported in wild birds (Zhao et al., 2014). In this study, two strains of the H9N2 AIV were isolated in 2014 from fecal samples of wild birds—one strain from Chen Lake in Hubei Province and one from Caizi Lake in Anhui Province of China-during avian influenza surveillance. Phylogenetic analysis revealed high homology of all six internal genes of these two strains with the internal genes of the H10N8 virus A/Jiangxi/Donghu-346/2013(H10N8) (referred to as JX346), which had caused lethal infection in humans in Jiangxi Province, China, in 2013. To better understand the biological characteristics of these two H9N2 virus strains, we analyzed special amino acids in the genome that may be associated with infectivity and pathogenicity. Furthermore, we assessed the virus replication capacity of the strains in mammals through experimental infections in mice. The results of this study suggest that these H9N2 AIVs from wild birds pose a potential threat to public health. In particular, the role of these AIVs as donors of internal genes cannot be ignored. AIV surveillance of wild birds needs to be strengthened to provide scientific evidence for use in the early warning and prevention of influenza viruses in poultry.

## Materials and methods

### Ethics statement

This study was carried out in strict according to the recommendations in the Guide for the Care and Use of Laboratory Animals of the Ministry of Science and Technology of the People's Republic of China. All animal experiments were preceded in the enhanced animal biosafety level 2+ (ABSL 2+) considerations in the Harbin veterinary research institute of the Chinese academy of agriculture science.

### Virus isolation and identification

Fecal samples from wild birds were collected at Chen Lake in Hubei Province in January 2014 and from Caizi Lake in Anhui Province in December 2014. The total sample size was 3710, including 740 samples from Chen Lake and 2970 samples from Caizi Lake. Bird species were identified by telescope observation before sampling, and the bird source of the fecal samples was determined on the basis of fecal shape and color. The samples were placed in a preservative solution containing penicillin-streptomycin, 30% glycerol and stored at −80°C in an ultralow-temperature freezer. During transportation, we put the samples in foam boxes with some ultralow temperature biological ice packs to maintain a low temperature and avoid thawing.

After preparation, the samples were inoculated into 9–11-day-old specific-pathogen-free (SPF) chicken embryos, and allantoic fluid was harvested after 72 h of culture. The culture was serially passaged three times following the same procedure which inoculated the harvested allantoic into 9–11 days old SPF eggs and culture 3 days. Hemagglutination activity (HA) was evaluated for each generation of allantoic fluid harvested by the HA test. Then, influenza virus and HA subtypes were identified by the hemagglutination inhibition (HI) test. Finally, the results were verified by subtype-specific reverse transcription PCR (RT-PCR) (Table 1). Reverse transcription primer of AIV used 12 bp primer, the sequence is 5′- AGC RAA AGC AGG-3′. Neuraminidase (NA) subtypes were directly analyzed by the subtype-specific RT-PCR and sequencing analysis. Ampilcation and sequencing of AIV' genome were according to the protocol as describe previously (Li et al., 2005).

**Table 1:** **Primers of amplification**.

**Primers name**	**Sequences**
H9HA-F	TTAGTAGAAACAAGGGTTTTTGCCAA
H9HA-R	TTAGTAGAAACAAGGGTTTTTGCCAA
N2NA-F	CCAGCAAAAGCAGGAGTAAAAATGA
N2NA-R	TTAGTAGAAACAAGGGAGTTTTTTCTAAA

### Phylogenetic analysis

All reference sequences used in this study were sourced from the NCBI GenBank database and the Global Initiative on Sharing All Influenza Data (GISAID). Sequence homology and key amino acid site analysis was performed using DNASTAR's MegAlign. Phylogenetic trees were constructed using MEGA6.0 with the Maximum likelihood algorithm (bootstrap values of 1000). No.KM076701-KM076708 in Genbank is HuB/S428 and KT699053-KT699060 is AH/L139.

### Mouse infection experiments

Six-week-old female BALB/c mice were purchased from the Experimental Animal Center of Vital River (Beijing, China). These mice randomly divided into two infection groups (eight mice/group) and one control group (five mice/group). Animals of the infection groups were mildly anesthetized with CO_2_ and inoculated with viruses by intranasal at a dose of 10^6^ EID_50_ (50 μl/mouse). Three days later, three mice were randomly selected from the infected group and euthanized with CO_2_ (Deng et al., 2015; Liang et al., 2015). Mouse organs, including the brain, turbinate, spleen, lungs, and kidneys, were collected and homogenized separately. The homogenates were used for virus titration in 9–11 days chick embryos. The virus titers were calculated by the method of Reed and Muench (Liang et al., 2015). The behavior and the body weight changes of the mice were observed and recorded for 14 consecutive days.

## Results

### Virus isolation, identification, and preliminary sequence analysis

In this study, 35 strains of virus were isolated from the allantoic fluid with HA activity from 3710 fecal samples. The HA-HI tests and PCR identified 32 isolates of influenza virus and three isolates of Newcastle disease virus. The 32 isolates of influenza virus contained eight subtype combinations: H1N1, H1N2, H3N3, H3N8, H6N1, H6N2, H9N2, and H11N9. On the basis of whole-genome sequencing, we found that all six internal genes of the two H9N2 AIV strains A/*Anser fabalis*/China/HuBS428/2014(H9N2) and A/*Anser fabalis*/China/Anhui L139/2014(H9N2) (HuB/S428 and AH/L139 for short) shared high homology with the internal genes of JX346, i.e., the human H10N8 virus reported in Jiangxi in 2013. These two H9N2 AIVs were isolated from Chen Lake in Hubei and Caizi Lake in Anhui. According to species identification before sampling and fecal morphology, these two strains were both collected from the bean goose (*Anser fabalis)*.

### Phylogenetic analysis

**Figure 1:**
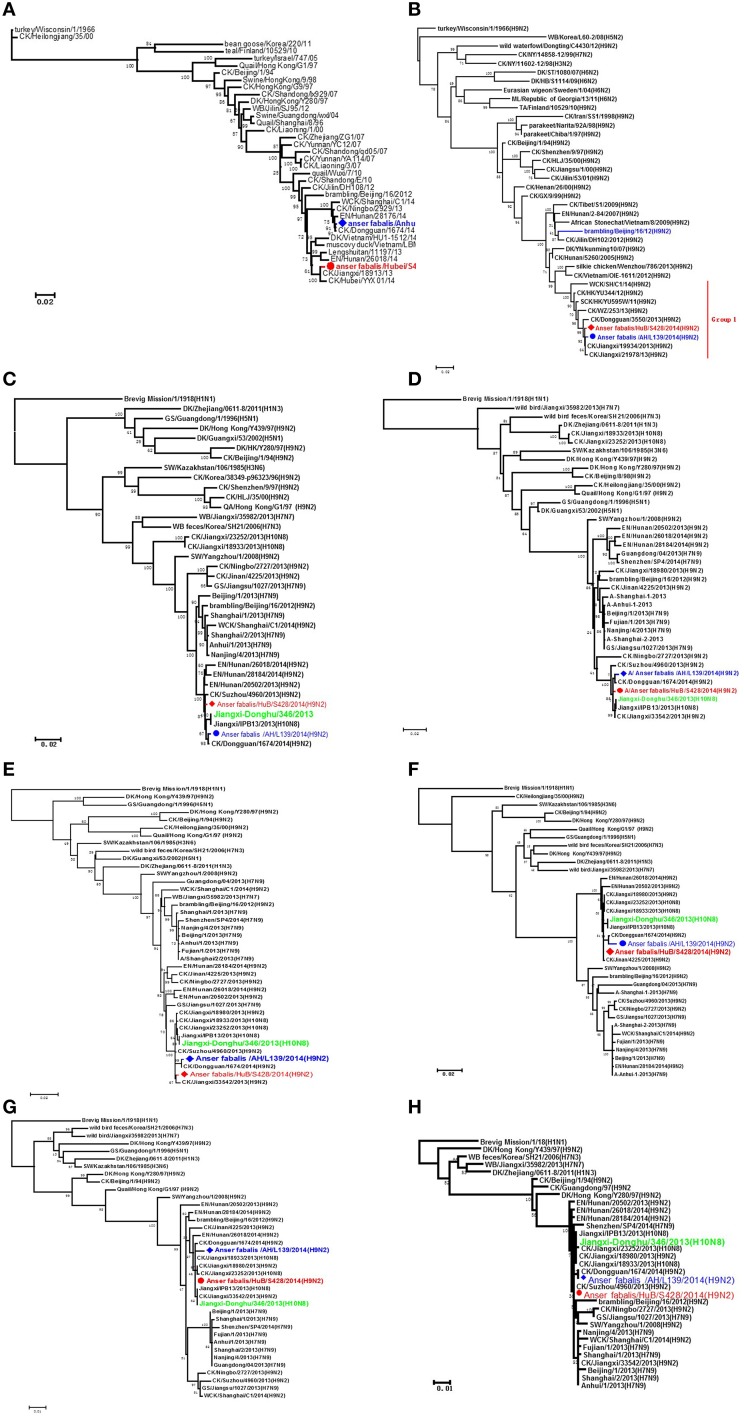
**Phylogenetic analyses of the H9N2 viruses isolated in this study**. The phylogenetic trees were generated with the PHYLIP program of the ClustaIX software package (version 1.81). The phylogenetic trees of HA **(A)** and N2 NA **(B)** were rooted to A/Turkey/Wisconsin/1/1966 (H9N2). The phylogenetic trees of PB2 **(C)**, PB1 **(D)**, PA **(E)**, NP **(F)**, M **(G)**, and NS **(H)** were rooted to A/Brevig Mission/1/1918(H1N1). Sequences of viruses with names in black were downloaded from available databases; viruses with names in red and blue were sequenced in this study. The colors of the virus names in the NA, PB2, PB1, PA, NP, M, and NS trees match with those used in the HA tree. CK, chicken; DK, duck, GS, goose; GX, Guangxi; HK, Hong Kong; HuB, Hubei; AH, Anhui; MDK, Muscovy duck; ML, mallard; SW, swine; WDK, wild duck; EN, environment.

Phylogenetic analysis showed that when various genes were compared between strains HuB/S428 and AH/L139, the HA gene shared a rather low homology of only 95.6% compared with other segments, whereas the other seven gene fragments shared high homology of more than 99%. Homology analysis with reference sequences from GenBank showed that the HA gene of strain HuB/S428 exhibited the highest sequence identity with A/chicken/Jiangxi/18913/2013(H9N2) (99.6%), whereas the same gene of strain AH/L139 is most homologous with A/chicken/Dongguan/1674/2014(H9N2) (up to 99.9%; Figure 1A). Genetic analysis showed that the NA gene is highly homologous between HuB/S428 and AH/L139 (up to 99.4%), only showing a difference of eight nucleotides and/or four amino acids in NA protein. Both strains share the highest homology with the NA gene of A/chicken/Jiangxi/19934/2013(H9N2) in GenBank (A: 99.8%; B: 99.6%; Figure 1B).

Strains HuB/S428 and AH/L139 are both highly homologous (>98.7%) with all the internal genes of the strain JX346 (H10N8), which had infected people in China (PA: 99.6 and 99.1%, PB1: 99.6 and 99.2%, PB2: 99.5 and 99.2%, NP: 99.7 and 98.7%, M: 99.9 and 99.3%, NS: 99.8 and 99.5%; Figures 1C–H). The internal genes of the novel H7N9 AIV that emerged in the Chinese population in 2013 reportedly are also derived from the H9N2 AIVs. In the present study, we compared internal genes between the two test strains and the first isolate of the human-infecting H7N9 AIV, A/Anhui/1/2013(H7N9). We found that HuB/S428 and AH/L139 are highly homologous with the AH/1(H7N9) AIVs and are assigned to the same branch (PB2: 98.3 and 98.0%, PB1: 97.6 and 97.4%, PA: 97.7 and 97.2%, NP: 96.2 and 95.3%, M: 98.0 and 97.4%, NS: 96.3 and 96.2%; Figures 1C–H). Individual homology analysis of the internal genes between the two test strains (HuB/S428 and AH/L139) and the reference strains (JX346 and Anhui/1) showed that HuB/S428 shares higher homology than AH/L139 with the references.

### Molecular characteristics

Strains HuB/S428 and AH/L139 both have a cleavage site for SRSS in HA protein, which is a characteristic of low pathogenic AIV in chicken. The hemagglutinin (HA) shows the characteristic of binding to the human 2, 6-NeuAcGal receptor at 226L and 228S (H3 numbering; Vines et al., 1998). The HA protein of HuB/S428 and AH/L139 shows the characteristic of 226L, suggesting that the two virus strains tend to bind to the human receptor. This result agrees with the reported capacity of the currently prevailing H9N2 AIV for binding to the human 2, 6 receptor, rather than reflecting the tendency of the earlier isolates, which more easily bound to the avian 2,3 receptor (Sun and Liu, 2015). Amino acid analysis of the NA gene revealed deletion of amino acids 63–65 in the neck of NA, indicating that HuB/S428 and AH/L139 have enhanced virus virulence in mice (Sun et al., 2010). The HA protein of HuB/S428 and AH/L139 have eight and seven potential N-X-T/S glycosylation sites (X is an amino acid other than P), respectively. These two strains both have a potential glycosylation site at position 313 for virus-mediated cell fusion and receptor-binding capacity (Kaverin et al., 2004).

Leucine (K) at 363 site in HA, and lysine (L) at 672 in PA which are considered to play an important role in the spread of the H9N2 virus in chickens in China (Zhong et al., 2014). No HA-K363 change was found in these two H9N2 AIV strains isolated in the present study. Nonetheless, their PA protein is identical to JX346 (H10N8) and Anhui/1(H7N9) at position 672, namely, lysine. Notably, both HuB/S428 and AH/L139, as well as JX346(H10N8) and Anhui/1(H7N9), have experienced a change in N383D in the PA protein (amino acid 383) which change at this site can enhance the virulence of the influenza virus (Song et al., 2011). The HA protein of both HuB/S428 and AH/L139 contains valine at position 368, and this characteristic can increase the transmissibility of the virus between ferrets (Herfst et al., 2012). Additionally, both HuB/S428 and AH/L139 contain PB2 (89V) (Li et al., 2009), M1 (N30D, T215A) (Fan et al., 2009), and NS1 (P42S) (Jiao et al., 2008), which can enhance virus virulence in mice. NS1 is associated with amino acid substitution of V149A, indicating that these two virus strains can antagonize the induction by interferon in chicken embryo fibroblasts (Li et al., 2004).

### Mouse infection experiments

**Figure 2:**
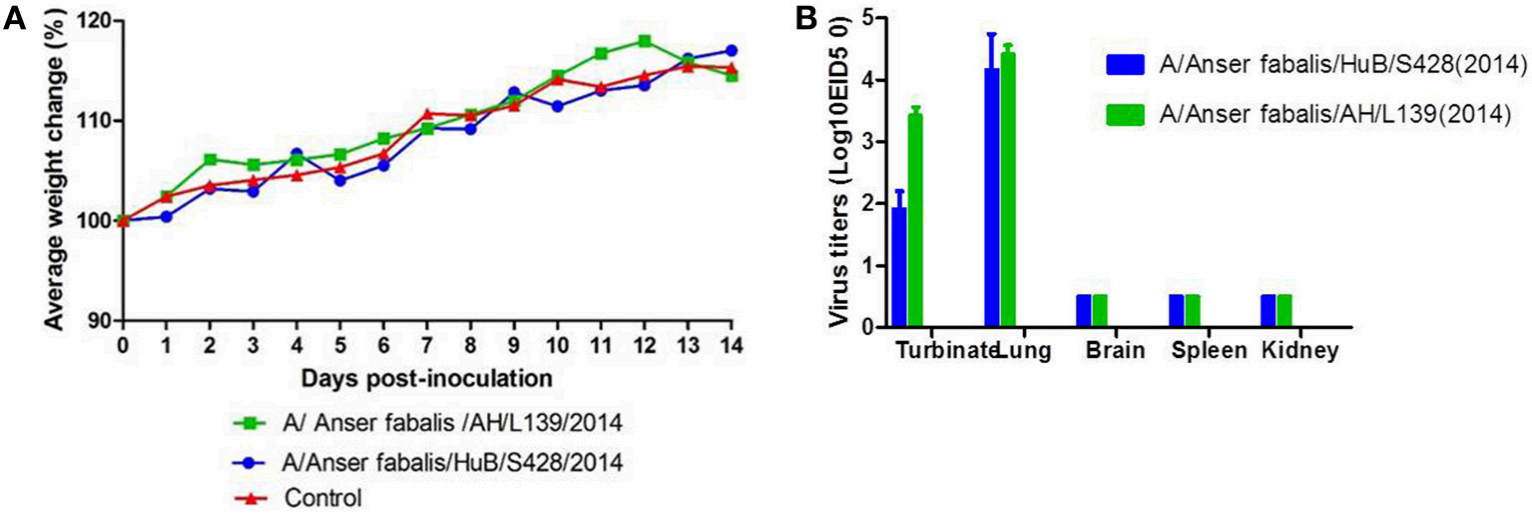
**Change in body weight (A) and mortality (B) in mice inoculated with strains A/Anserfabalis/HuB/S428(2014) and A/Anserfabalis/AH/L139(2014) of the H9N2 influenza viruses**. **(A)** Body weights and survival rates of mice were observed over 14 days after infection. **(B)** Coloums show the mean titers from three mice; Error bars indicated the standard deviations. Five organs were collected at 3 days postinfection (p.i.); the virus replication levels were measured by EID_50_ in specific-pathogen-free (SPF) eggs.

To evaluate the replication and virulence of the two virus strains to mammal, we used conventional methods to perform mouse infection experiments in Biosecurity level 2+ laboratory. After infection, the mouse body weight showed a transient decrease or temporarily ceased to grow and then immediately returned to normal growth. Mice maintained a stable body weight at days 2–5 after infection with strain AH/L139. The body weight of mice showed a transient decrease at days 4–6 after infection with strain HuB/S428 and then returned to normal growth; the maximum decrease was up to 2.52% (Figure 2A). All mice survived in observation days. Virus titration of the mouse organs showed that neither test strains replicated in the brain, spleen, or kidneys of the mice. Nonetheless, the mice had relatively high viral titers in the lungs: 4.17 ± 0.58 log10 EID_50_ for HuB/S428 and 4.41 ± 0.14 log10 EID_50_ for AH/L139. The viral titers of strains HuB/S428 and Anhui/L139 in the turbinate were 1.92 ± 0.29 and 3.42 ± 0.14 log10 EID_50_, respectively, indicating differential replication (Figure 2B). Virus titers were markedly higher in the lungs than in the turbinate.

## Discussion

Wild birds provide an influenza virus gene pool and play an important role in the genetics, evolution, and spread of viruses (Li et al., 2005). Since human infections with H7N9 and H10N8 AIVs emerged in China in 2013, many scholars have conducted extensive influenza virus surveillance of wild birds to better understand the role of wild birds in disease development and epidemics. However, except for one strain of H7N9 AIV isolated from a sparrow in Shanghai in 2014, no other reports are available on human H7N9 and H10N8 viruses in wild birds (Zhao et al., 2014). From the spring of 2013 to the spring of 2015, we collected approximately 20,000 fecal samples of wild birds from several provinces, including Hubei and Anhui. The H7N9 and H10N8 viruses were not detected in these samples (data not shown). Our surveillance data further support the inference that poultry, especially in the live poultry markets, are the primary source of the novel H7N9 and H10N8 viruses that infect humans (Ma et al., 2015).

Although we did not find H7N9 and H10N8 viruses in the surveillance of wild birds, we were interested in the two H9N2 virus strains (HuB/S428 and AH/L139) isolated from fecal samples of wild bean goose in Anhui and Hubei provinces in 2014. All six internal genes of strains HuB/S428 and AH/L139 are highly homologous with the internal genes of the human H10N8 virus, as well as with the human H7N9 virus. Hubei and Anhui are adjacent to Jiangxi Province, and human infection with H10N8 was reported in Jiangxi. Sequence comparison of surface genes revealed that these two virus strains are most homologous with the H9N2 virus from poultry in Jiangxi or Guangdong provinces and clustered with the H9N2 reference strain from poultry in South China. With regard to internal genes, strains HuB/S428 and AH/L139 are most homologous with H10N8 (Jiangxi) and H9N2 (Guangdong) in poultry. These results suggest that these two wild bird-derived H9N2 AIVs may be derived from poultry in South China. Avian influenza virus could had some mutation among evolution and reassortment, such as the character of receptor-binding, replicated ability in mammal, virulence, transmission and so on. The HA protein of both strains in this study has the characteristic of binding to human receptors (226L). The deletion of some amino acid sequence in NA protein in the neck, which can enhance the pathogenicity of these strains in mice. Furthermore, we compared the corresponding amino acid sequences of internal genes and found amino acid changes associated with pathogenicity, interferon antagonism, and transmissibility in the amino acid sequences of PB2, PB1, M1, NS1, and PA. Mouse infection experiments showed that both strains of the H9N2 AIVs from wild birds directly infected mice without adaptation. Additionally, higher virus titers were detected in the lungs and turbinate, indicating that these two virus strains potentially have the capacity to infect mammals directly.

H9N2 AIVs are widely distributed on the continents of the world; however, they mainly prevail in poultry in Asia and are some of the most widely prevalent AIVs in poultry in mainland China. The stable existence and persistent of the prevalence of H9N2 AIVs in poultry not only causes serious harm to the poultry industry but also constitutes an increasingly serious threat to human health. The extensive and stable presence of this subtype AIVs in poultry provides favorable conditions for the emergence of a novel virus with pathogenicity to humans in China.

Currently, the amino acid cleavage sites of the HA protein in human H10N8 and H7N9 viruses are known to have the characteristics of low pathogenicity in AIVs (Liu et al., 2013; Chen et al., 2014). However, both H10N8 and H7N9 viruses are able to infect humans and cause death. Notably, the entire internal gene sequences of both viruses share high homology and both originate from the H9N2 virus in poultry. This phenomenon suggests that this particular internal gene complex may have an important role in enhancing the infectivity and pathogenicity of the influenza virus in humans. That is, the “harmful” internal gene complex of the H9N2 virus from poultry might convey human infectivity and pathogenicity to these novel recombinant viruses.

Wild birds serve as a huge reservoir and effective communicator of influenza viruses, playing a significant role in promoting virus spread and recombination (Olsen et al., 2006), In particular when seasonal migration of migratory birds and their close contact with local sentinel chickens and/or ducks (Ma et al., 2015). Our findings prove the presence of H9N2 viruses in wild birds carrying internal genes that are highly homologous with human H10N8 and H7N9 viruses. Inner genes combination played the determinative role in AIV' transmissibility in mammal (Zhang et al., 2013b). Wild birds which containing a “harmful” internal gene complex, enabling the generation of novel pathogenic viruses by gene rearrangement with other influenza viruses, lethal to human health in the future. Therefore, strengthening the AIV surveillance of wild birds will enable understanding of the presence and prevalence of influenza viruses and provide scientific evidence for use in the prevention and control of AIVs.

## Author contributions

GY contributed on experiment, writing article. CL contributed on guiding the experiment, and revising the article. GY with him are co-sensor authors. HP, corresponding author, contributed on major guiding the experiment. DH contributed on giving a hand in experiment. LS, YX, and CG contributed on collecting the samples.

### Conflict of interest statement

The authors declare that the research was conducted in the absence of any commercial or financial relationships that could be construed as a potential conflict of interest.
